# Safety and effectiveness of delivering mass drug administration for helminths through the seasonal malaria chemoprevention platform among Senegalese children: study protocol for a randomised controlled trial

**DOI:** 10.1186/s13063-022-06579-0

**Published:** 2022-08-03

**Authors:** Muhammed O. Afolabi, Doudou Sow, Jean Louis A. Ndiaye, Brian Greenwood

**Affiliations:** 1 Department of Disease Control, London School of Hygiene & Tropical Medicine, London, UK; 2 Université Gaston Berger de Saint-Louis, Saint-Louis, Senegal; 3 Université de Thies, Thies, Senegal

**Keywords:** Malaria, Geo-helminths, Schistosomiasis, Children, Africa, Randomised controlled trial

## Abstract

**Background:**

Malaria remains a major health problem, especially in sub-Saharan Africa where more than 90% of the disease and where nearly all deaths occur in children. Adding to this high burden is the co-existence of intestinal and genito-urinary helminth infections. Existing control programmes for these helminths are operating sub-optimally. Conversely, a malaria prevention programme, called seasonal malaria chemoprevention (SMC), introduced in 2012 has achieved more than 75% treatment coverage and prevented 75–85% cases of uncomplicated and severe malaria in children. This encouraging development supports the need to explore strategies involving the integration of helminth control with successful platforms such as SMC. This would align worm and malaria control within the Sustainable Development Goals of ending the diseases of poverty and promoting health and well-being for those at risk.

**Methods:**

This study will have quantitative and qualitative components. The quantitative component will be a three-arm, observer-blind, placebo-controlled, interventional study of co-administration of SMC and anthelminthic drugs to pre-school and school-age children in Saraya district, southeast Senegal. Six hundred children aged 1–14 years will be randomly assigned to receive either SMC drugs only, SMC drugs and praziquantel or SMC drugs and albendazole and praziquantel at a ratio of 1:1:1. The primary outcome will be solicited and unsolicited adverse reactions to the study medications. The secondary outcomes will be the prevalence and intensity of *Plasmodium*-helminth co-infection and the prevalence of anaemia and mean haemoglobin concentration.

The qualitative component of the study will include the conduct of structured interviews to assess the acceptability, feasibility, enablers and barriers to the combined use of anthelminthic and SMC drugs among randomly selected parents/caregivers of children enrolled in the study and health care workers responsible for the delivery of the combined services.

**Discussion:**

This study will provide evidence to boost the public health recommendations for combined malaria and helminth control. If successful, this project will reinforce the evidence that health care systems in developing countries can be comprehensive health management rather than focussed on vertical management of a single disease.

**Trial registration:**

ClinicalTrials.gov NCT05354258. Registered on 28 April 2022. PACTR202204794105273. Registered on 25 April 2022

## Administrative information

Note: The numbers in curly brackets in this protocol refer to SPIRIT checklist item numbers. The order of the items has been modified to group similar items (see http://www.equator-network.org/reporting-guidelines/spirit-2013-statement-defining-standard-protocol-items-for-clinical-trials/).

**Table:** 

Title {1}	Safety and effectiveness of delivering mass drug administration for helminths through the seasonal malaria chemoprevention platform among Senegalese children (MALHELMIN study)
Trial registration {2a and 2b}.	Clinical Trial.gov NCT05354258 PACTR202204794105273
Protocol version {3}	Version 2.0, 8 April 2022
Funding {4}	UK Research and Innovation (UKRI) Future Leaders Fellowship awarded to MOA, grant reference number MR/S03286X/1
Author details {5a}	Muhammed O. Afolabi^1^, Doudou Sow^2^, Jean Louis A. Ndiaye^3^, Brian Greenwood^1^ ^1^London School of Hygiene & Tropical Medicine, London, United Kingdom ^2^Université Gaston Berger de Saint-Louis, Senegal ^3^Université de Thies, Thies, Senegal
Name and contact information for the trial sponsor {5b}	Patricia Henley Head, Research Governance & Integrity Office London School of Hygiene & Tropical Medicine, Keppel Street, WC1E 7HT, London, United Kingdom E-mail: Patricia.Henley@lshtm.ac.uk
Role of sponsor {5c}	The first author (MOA) is a member of the academic staff of the London School of Hygiene & Tropical Medicine (Study sponsor) and has the ultimate responsibility for the study design; data collection, study management, data analysis, and interpretation of data; writing of the report; and the decision to submit the report for publication. The funder has no role in the study design, data collection and analysis, decision to publish, or preparation of the protocol.

## Introduction

### Background and rationale {6a}

Malaria and helminths frequently co-habit in children living in sub-Saharan Africa (SSA). An interplay of environmental and host factors favours mixed infections of malaria species with parasitic helminths including soil-transmitted helminths (STH) and *Schistosoma* species [1, 2]. Statistical and spatial models support the geographic overlap and co-endemicity of falciparum malaria and hookworm infections in SSA [3]. Similar spatial distribution studies have documented the association between malaria and schistosomiasis [4].

Despite the considerable progress that has been made in the control of malaria, SSA still account for an estimated 228 million cases in 2020, representing about 95% of global malaria cases [5]. Similar progress has been reported in the control of neglected tropical diseases (NTDs), chief among which are STH and schitosomiasis, which, however, still impose a major human, social and economic burden on more than 1 billion people globally, predominantly among vulnerable paediatric populations in SSA. It is estimated that 1.5 billion individuals are infected with helminths globally [6], with more than 800 million children in SSA affected by STH primarily hookworm (*Ancylostoma duodenale* and *Necator americanus*), roundworm (*Ascaris lumbricoides*) and whipworm (*Trichuris trichiura*) [7]. Other important helminths that co-exist with malaria in children in SSA include *Schistosoma haematobium* and *S. mansoni* [8].

The geographical overlap in the co-endemicity of helminths and malaria parasites may result in synergistic or antagonistic interactions between helminths and malaria parasites [2, 9, 10]. There is some evidence that infections with *Schistosomes* and STH exert deleterious effects on the course and outcome of clinical malaria [3, 9]. Prominent among the clinical outcomes of malaria-helminth co-infections among children living in SSA is anaemia [11, 12] resulting from blood loss, haemolysis, inflammatory processes or splenic sequestration [13, 14]. The additive effects of malaria-helminth co-infection on haemoglobin concentration probably contribute significantly to mortality from malaria in children in SSA [11, 15]. Also, a substantial proportion of malaria mortality in children is linked to anaemia [16], while iron deficiency anaemia (IDA) induced by helminth infestations is associated with stunted growth, impaired cognitive development, poor school attendance and low academic performance [15, 16].

Given this context, it is important to develop a treatment approach that combines malaria and helminth control in an integrated framework that is safe, effective and easy to deliver. This study will investigate the feasibility and effectiveness of co-administration of anthelminthic and SMC drugs in a high-risk paediatric population living in a malaria-helminth co-endemic setting of Senegal, West Africa. This study is designed to test the hypothesis that co-administration of SMC and anthelminthic drugs will be safe and tolerated among children aged 1–14 years and that the incidence of side effects will not be significantly higher in children who receive the combined SMC and anthelminthic drugs than in those who receive SMC alone. The study will also seek to establish the extent to which combined administration of SMC and anthelminthic drugs will reduce the prevalence of anaemia, malaria and schistosomiasis compared to SMC administration alone.

Given that the feasibility of delivery and the effectiveness of an intervention may be influenced by its acceptability by end-users and other stakeholders, the perceptions of parents/caregivers of children enrolled in this study, SMC providers and programme managers will be explored to gain information on the acceptability, feasibility, barriers, enablers and practicalities of using the integrated SMC-anthelminthic approach.

Overall, this study may provide evidence to boost public health recommendations for combined malaria and helminth control. If successful, this project will reinforce the evidence that the future direction of health care systems in developing countries should be comprehensive rather than vertical management of a single disease.

### Objectives {7}

The primary objective of this study is to assess the safety and tolerability of concurrent administration of SMC and anthelminthic drugs among pre-school and school-aged children in Senegal. The secondary objective is to evaluate the effects of combined treatment with SMC and anthelminthic drugs on the prevalence of malaria, anaemia and helminth infections among pre-school and school-age children in Senegal. The qualitative component of this study will explore the perceptions of parents/caregivers of study children, SMC/NTD providers and programme managers about the acceptability, feasibility, barriers, enablers and practicalities of using the integrated SMC-anthelminthic approach.

### Trial design {8}

**Fig. 1:**
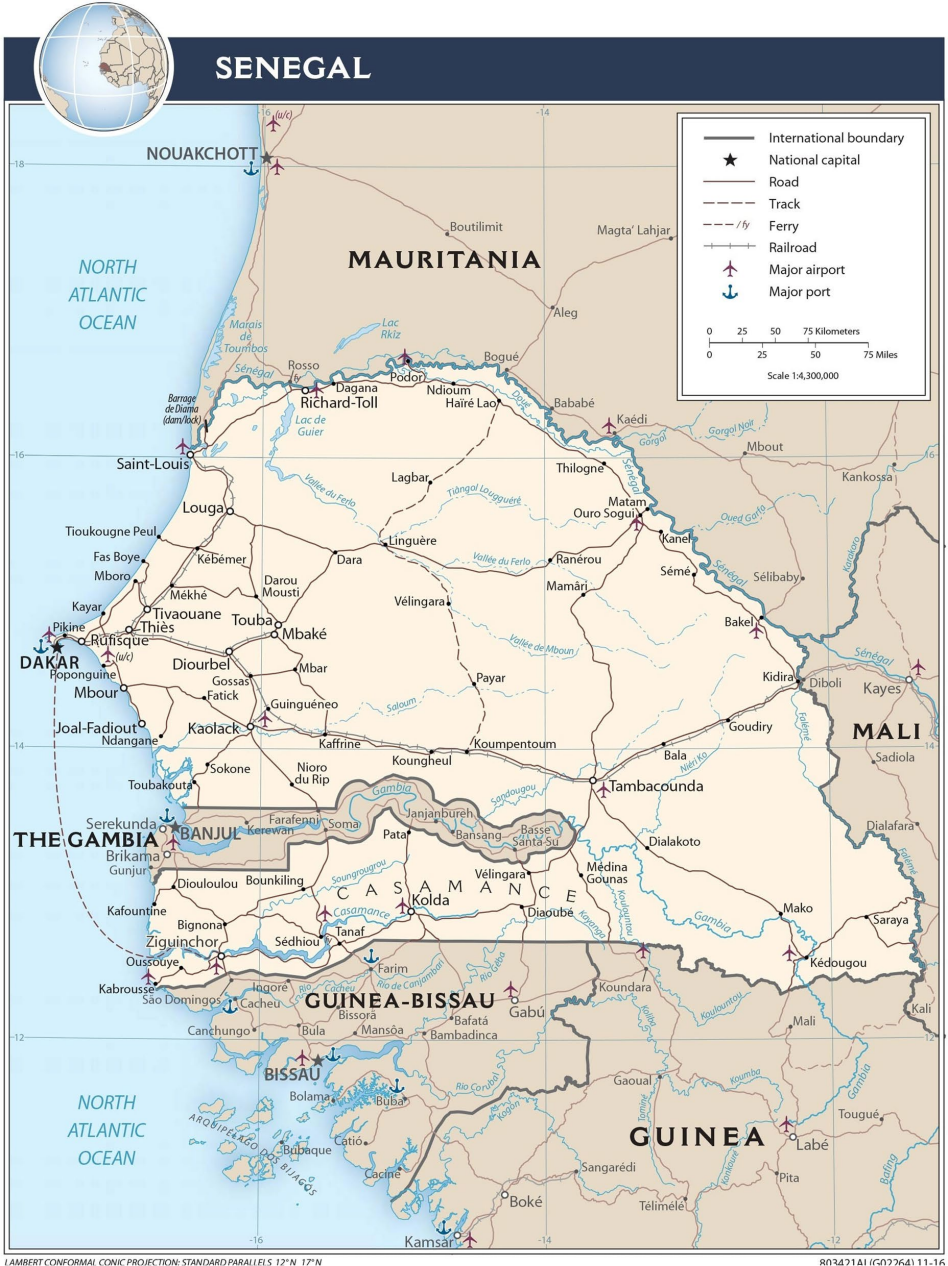
Map of Senegal showing the study site. Source: https://www.cia.gov/resources/map/senegal/. Terms of use: https://www.cia.gov/site-policies

A non-inferiority trial design is adopted to demonstrate that integrated SMC and anthelminthic drug delivery will be safe and tolerated among the study participants and that the incidence of the side effects will not be significantly higher in the study participants who receive the combined SMC and anthelminthic drugs than in those who receive SMC drugs alone. The study will include quantitative and qualitative components. The quantitative component will be a three-arm, observer-blind, placebo-controlled, interventional study of concurrent administration of SMC and anthelminthic drugs to pre-school and school-age children in the Saraya district of Kedougou region, southeast Senegal (Fig. 1).

**Fig. 2:**
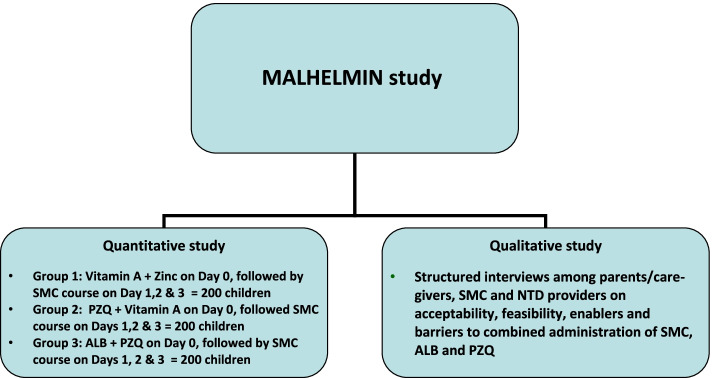
Schematic diagram of the study design: A day before the commencement of the first cycle of SMC, 600 eligible children aged 1–14 years will be randomly assigned to one of three arms at a ratio of 1:1:1 to determine the safety, feasibility and tolerability of the SMC and anthelminthic drug co-administration. The first group will receive vitamin A and zinc supplement and the second group will receive praziquantel and vitamin A while the third group will receive albendazole and praziquantel. On the following day, corresponding to the start (day 1) of the first SMC cycle, all the three groups will receive amodiaquine (AQ) and sulphadoxine/pyrimethamine (SP) according to the WHO and Senegal SMC implementation guidelines. On the second and third day of the SMC cycle, all study children in the three groups will receive AQ, in line with the SMC guidelines

In June 2021, prior to the start of the malaria transmission season and before SMC administration commenced, we conducted a population-based survey to estimate the prevalence of malaria-helminth co-infections among children aged 1–14 years in 18 villages in Saraya district. Preliminary findings of this survey showed that the prevalence of malaria ranged between communities from 3.3 to 9.5% and of *Schistosome* infection from 12.2 to 64.8% (unpublished data). These findings will guide the selection of the villages for implementing the study described in this protocol. The schematic diagram of the study design is illustrated in Fig. 2.

The qualitative component of the study will involve the conduct of structured interviews to assess the acceptability, feasibility, enablers and barriers to the combined use of anthelminthic and SMC drugs among randomly selected parents/caregivers of children enrolled in the study. Key informant interviews will also be held with SMC and MDA (anthelminthic) providers as well as with the SMC and NTD programme managers from the Senegal Ministry of Health to further explore these factors.

## Methods: participants, interventions and outcomes

### Study setting {9}

The study will take place in villages in Saraya district, Senegal. Saraya district borders Mali to the east and Guinea to the south and is composed of the rural communities of Sabadola, Khossanto, Bembou, Missirah Dantilla, Missirah Sirimana and Medina Baffe and includes 102 villages occupying a land area of 7803 km^2^, with an estimated total population of 61,756 people, including 10, 566 under-five children and 5,770 children aged between 5 and 10 years. The Saraya department has one health centre, 10 health posts, two health huts and 39 home care providers (2019 population census estimate) (Fig. 1). Over 70% of the population live more than 15 km from a health facility. The main ethnic groups are Malinke and Diakhanke. The climate is Sudano-Sahelian with two seasons, a dry season from November to May and a rainy season from May to November. The average rainfall ranges from 16 to 4mm during these months, with an average rainfall of 213 mm during the peak period in August. Mean daily temperatures range from 14 to 36°C from July to February and 21–40°C from March to June [17]. Saraya district is one of the zones in Senegal most affected by both malaria [18] and helminths [19].

### Eligibility criteria {10}

#### Inclusion criteria

- Male or female children aged 1–14 years
- Provision of written informed consent by the parent/caregiver and a positive assent by children aged ≥ 12 years (in line with legal regulations in Senegal)
- Willingness to provide finger-prick blood samples and urine and stool samples
- Residence in the study area for at least six months

#### Exclusion criteria

- Acutely ill child at the time of the drug administration
- A child whose parents/caregivers decline to provide consent
- A known HIV-positive child receiving cotrimoxazole prophylaxis
- A child who has received a dose of either sulphadoxine-pyrimethamine (SP), amodiaquine (AQ), albendazole (ALB) or praziquantel (PZQ) during the previous six  months
- A child with a known allergy to any of SP, AQ, ALB or PZQ

### Who will take informed consent? {26a}

Written informed consent will be obtained from potential trial participants by a trained research assistant. The Participant Information Sheet will be translated to French and a trained field assistant who is a native speaker will interpret the information in a Senegalese language preferred by the parents/caregivers. If the parent/caregiver is not able to read and write in French, a literate adult witness will be present throughout the whole consent process and sign and date the consent form. A parent/caregiver must provide written informed consent form, before a child aged <12 years is enrolled into the study. For children aged ≥ 12 years, positive assent is required in addition to the parental consent.

### Additional consent provisions for the collection and use of participant data and biological specimens {26b}

Additional consent provisions for the collection and use of participant data and biological specimens in future studies will be obtained by a trained research assistant during the consent discussion.

### Interventions

#### Explanation for the choice of comparators {6b}

To ensure objectivity in the reporting of adverse events among the study children, vitamin A and a zinc supplement will be used as controls, so that all children randomised to each study arm receive three drugs [20, 21]. Vitamin A will be an oral liquid, oil-based preparation of retinyl palmitate or retinyl acetate, obtained from the UNICEF, Senegal office. The zinc preparation will be a citrus-flavoured tablet containing 25 mg Zn in the form of zinc sulfate and will be obtained from Biolectra Zink; Hermes Arzneimittel GmbH, Munich, Germany. To minimise under- and over-dosing while keeping simple dosage recommendations, the doses of vitamin A and zinc supplement will be based on the child’s age, as follows: children aged 1–14 years will receive 200,000 IU vitamin A and zinc (40 mg/kg) and zinc supplement (40mg/kg) based on their body weight.

#### Intervention description {11a}

Given that this study will be implemented during SMC campaign and in collaboration with the SMC programme of the Senegal Ministry of Health, the SMC drugs: AQ and SP will be obtained from the SMC implementation unit of the Ministry. The albendazole [ALB] and praziquantel [PZQ] needed for this study will be procured from GlaxoSmithKline and Merck, respectively. The doses of SP and AQ for SMC and anthelminthic drugs will be based on the child’s age in Table 1.

**Table 1:** Dosages of SMC and anthelminthic drugs to be used in this study

**SP tablet (500mg + 25mg)**	
• Children aged 12–59 months will receive a full tablet as a single dose on the first day	
• Children aged 5–10 years will receive one and a half tablet as a single dose on the first day	
• Children aged 11–14 years will receive two tablets as a single dose on the first day	
**AQ tablet (153mg base)**	
• Children aged 12–59 months will receive a full tablet as a single daily dose for 3 consecutive days	
• Children aged 5–10 years will receive one and a half tablet as a single dose for 3 consecutive days	
• Children aged 11–14 years will receive two tablets as a single dose for 3 consecutive days	
**ALB tablet (200 or 400 mg depending on age)**	
• Children aged 12–24 months will receive a half dose of albendazole 200mg	
• Children aged greater than 2 years and up to 14 years will receive a single dose of albendazole 400mg	
**PZQ tablet (40 mg/kg)**	
• Children aged 1–14 years will receive praziquantel based on their body weight 40mg/kg	

#### Control

To ensure objectivity in the reporting of adverse events among the study children, vitamin A and zinc supplement will be used as control drugs, so that the children randomised to all study arms receive three drugs. Vitamin A and zinc supplements have not been shown to affect the outcomes of this study [20, 21].

#### Criteria for discontinuing or modifying allocated interventions {11b}

There will be no special criteria for discontinuing or modifying allocated interventions.

### Strategies to improve adherence to interventions {11c}

Given that the side effects caused by interactions of praziquantel with albendazole are dose-dependent [22, 23] and can be reduced by taking praziquantel with carbohydrate meals [24], we will give praziquantel to the study children based on the dose recommended by WHO (40mg/kg) and we will ensure that the children take a carbohydrate-rich meal before administration of the drugs. A trained member of the research team will visit the children at home to administer the drugs in the presence of their parents/caregivers. Children who start treatment but who could not be found at home after reasonable efforts will be excluded from the study. A simple, user-friendly recording tool will be used to capture the drug administration for each child.

### Relevant concomitant care permitted or prohibited during the trial {11d}

Implementing the concurrent administration of SMC and anthelminthic medications in this trial will not require alteration to the usual care pathways (including use of any medication) and these will continue for all trial arms.

#### Provisions for post-trial care {30}

There is no anticipated harm and compensation for trial participation.

### Outcomes {12}

#### Primary outcome

- All solicited and unsolicited adverse events will be assessed for causal relationships to the study medications, for six consecutive days after the administration of anthelminthic drugs.

#### Secondary outcomes

- Prevalence and intensity of *Plasmodium*-helminth co-infection
  - Faecal egg counts for each of the four parasites (hookworm, *A. lumbricoides*, *T. trichiura*, *and S. mansoni*) and urine egg count for *S. haematobium* will be recorded, and the prevalence and arithmetic mean intensity of *Plasmodium* infection will be calculated before and after concurrent administration of SMC and anthelminthic drugs (i.e. at the end of malaria transmission season).
- Prevalence of anaemia and mean haemoglobin concentration
  - Haemoglobin concentration of all children will be checked using a HemoCue® haemoglobin analyser, before and after co-administration of SMC and anthelminthic drugs (i.e. at the end of malaria transmission season).

### Participant timeline {13}

#### Sample size {14}

Haemoglobin (Hb) concentration was used to calculate the sample size. Given that the mean Hb concentration in the SMC alone group in a previous study [18] in the same population was 10.5 g/dl, we assumed a mean Hb concentration of 11.0 g/dl in the SMC + anthelminth arm and a SD of the Hb concentration of 1.5 g/dl. To detect a mean difference between the treatment arms of 0.5 g/dl with 90% power at a 95% level of statistical significance, a minimum of 189 children per treatment arm will be needed. Allowing for 10% loss to follow-up, approximately 200 children will be enrolled into each treatment arm.

Based on a recent survey conducted in the study site (unpublished data), we assumed that the prevalence of any malaria or helminth infection in the SMC group will be 30%, the prevalence of any malaria or helminth infection in the SMC + anthelminth arms will be 16% and the relative risk will be 0.53 (that is, 47% reduction in the SMC + anthelminth arms compared to the SMC alone group). A minimum sample size of 188 children was considered sufficient to give 90% power at 95% confidence interval and 5% level of precision. Adding 10% loss to follow-up, approximately 200 children will be needed for each treatment arm. Therefore, a total of 600 children aged 1–14 years will be enrolled and randomly assigned at a ratio of 1:1:1 into the control and the two intervention arms.

### Recruitment {15}

#### Stakeholder engagement and community sensitisation

The PI and co-investigators in Senegal have held several meetings with the programme managers and national coordinators of the SMC and NTD programmes of the Senegal Ministry of Health about this study, and these officers have provided their support during the implementation of the first stage of this project involving two population-based surveys in 2021. The study team will meet again with the community leaders and other stakeholders in the host community. Community sensitisation and engagement meetings will be organised prior to the commencement of the study to explain the nature of the study to the parents/caregivers of potentially eligible children*.*

During these meetings, the study team will explain the need for this study, using a simple picture to illustrate the concurrent administration of SMC and anthelminthic drugs; the study rationale and informed consent procedure; and the risks and benefits of allowing their children to participate in the study. After these meetings, trained research staff will identify parents/caregivers of potential study participants to explain the study further to them on an individual basis. Parents/caregivers who feel that the study is appropriate for their child will be visited at home at a mutually agreed time. For logistical purpose, consent will be obtained from a parent/caregiver one day before the planned start date of the study. A research staff member will provide a pre-labelled stool collection cup to the parent/caregiver at this visit and encourage them to collect stool from their child and keep same safely for the research team who will visit them, the following day.

### Assignment of interventions: allocation

#### Sequence generation {16a}

Randomisation will be used to minimise bias in the assignment of eligible children to the study arms to increase the likelihood that known and unknown attributes (e.g. demographic and baseline characteristics) are evenly balanced across arms and to enhance the validity of possible statistical comparisons across arms. Randomisation will be performed by a study pharmacist in blocks of 3 using a table of random numbers and will be stratified by age groups.

#### Concealment mechanism {16b}

This is not applicable, because the parents or caregivers of the children will probably know which drugs their children receive during the SMC campaign.

#### Implementation {16c}

The allocation sequence will be generated by a study pharmacist. The study participants will be enrolled by trained research assistants who will assign the participants to the treatment arms.

### Assignment of interventions: blinding

#### Who will be blinded {17a}

To minimise bias, the study staff who will administer the drugs to the children will be a completely separate group of staff from those who will conduct safety assessment for the study participants. This group of study staff who will perform the safety assessments will be blinded to the treatment arms. The data analyst will not be masked to the sources of the data.

#### Procedure for unblinding if needed {17b}

The design is open label with only outcome assessors being blinded, so unblinding will not occur.

### Data collection and management

#### Plans for assessment and collection of outcomes {18a}

On the start day of the study, the research staff will confirm the parent/caregiver’s willingness to allow their child’s continued participation in the study and administer a purpose-designed electronic questionnaire to the parents/caregivers. The questionnaire will cover information such as socio-demographic, health and residence characteristics, history of de-worming and prior malaria treatment. Height/length (cm) and weight (kg) of each child will be measured and anthropometric indices, height-for-age, weight-for-age, weight-for-height and body mass index, will be calculated using the WHO AnthroPlus software (www.who.int/growthref/tools/en/). The study villages will be mapped by recording each study participant’s household global positioning system (GPS) coordinates with a hand-held GPS device.

The quantitative study will be implemented in collaboration with the SMC and NTD providers during the SMC campaign which will start in Saraya district by the middle of June 2022. Two hundred children pre-school and school-age children will be enrolled into each arm of the study as described above. The study procedures are illustrated in Fig. 2 and Table 2. The children will be evaluated one  month after the last course of SMC cycles at the end of malaria transmission season to measure haemoglobin concentration and to obtain a further blood film, dried blood spot, stool and urine samples.

**Table 2:** Schematic diagram of the study procedures

SMC cycle	1					2, 3 and 4	End of malaria season
Timelines (days)	Day 0–1	Day 0	Day 1	Days 2 and 3	Days 4–6		
**Study procedures**						**Passive surveillance**	
Informed consent ± assent	X						
Enrolment and questionnaire administration to parent/caregivers		X					
Distribution of collecting bottles for stool sample	X						
Sample collection (finger-prick blood, urine, stool)		X					
**Randomisation**		X					
Group 1: vitamin A + zinc		X	AQ + SP	AQ			
Group 2: PZQ + vitamin A		X	AQ + SP	AQ			
Group 3: ALB + PZQ		X	AQ + SP	AQ			
Safety assessments		X	X	X	X		
**Post-intervention survey**							X
Sample collection: finger-prick							X
Sample collection: stool							X
Sample collection: urine							X

Qualitative interview participants will be 50 randomly selected parents/caregivers of study children, 10 SMC/MDA implementation staff and six SMC/NTD programme managers. The participants will be segregated by gender to allow free expression of views within each homogenous group. A purpose-designed guide will be used to facilitate these sessions. The proceedings of the sessions will be audio-recorded following verbal consent by the participants. These will be transcribed into texts by trained research assistants.

#### Plans to promote participant retention and complete follow-up {18b}

A reminder system will be implemented using short text messages (SMS) delivered via mobile telephones to all health centres staff reminding them to regularly look out for the study participants, asking them to report any serious events. A phone credit will be provided to the staff as an incentive, and they will be contacted after each SMC round to confirm whether they have observed any adverse events. The health post staff will be linked to a member of the study team who will contact them by phone or in person throughout the study to maintain contact, ask about any problems, give support and advice and remind them about study procedures using a standardised list of reminders.

#### Data management {19}

All data will be collected using encrypted android tablets and subsequently transferred to the University of Thies’ encrypted RedCap server in Senegal. Following export, they will be held on LSHTM’s network drive (in a personal area restricted to the project team) and accessed using encrypted laptops. All data generated in this project will be managed in accordance with the LSHTM Information Management and Security Policy, which has been drawn up in line with ISO/IEC 27001 and other relevant legal, procedural and technological requirements.

#### Confidentiality {27}

All study children will be assigned a unique number, which will be used as an identifier throughout the study. Participant data will be securely stored in the dedicated study’s computer devices, which will be accessible only with a password. All laboratory results and adverse event data will be encoded in an electronic database and stored securely by the PI or designee. Only authorised study personnel will have access to the study data.

The collection and processing of personal data from the participants enrolled in this study will be limited to those data that are necessary to fulfil the objectives of the study. These data will be collected and processed with adequate precautions to ensure confidentiality and compliance with applicable data privacy protection laws and regulations. Appropriate technical and organisational measures to protect the personal data against unauthorised disclosures or access, accidental or unlawful destruction, or accidental loss or alteration will be put in place.

#### Plans for collection, laboratory evaluation and storage of biological specimens for genetic or molecular analysis in this trial/future use {33}

Prior to the administration of study drugs, finger-prick blood samples will be collected from the participants for preparation of thick and thin blood smears for malaria microscopy, and blood spots will be collected on Whatman filter papers for DNA isolation and PCR amplification for species determination. Freshly voided urine samples will also be collected from all study children. The urine filtration test will be used to quantify *S. haemotobium*, according to WHO guidelines. Parallel testing for circulating cathodic antigens (CCA) will be used to complement the filtration test [25]. The urine CCA dipstick test has been extensively used in SSA and has sensitivity and specificity values for *S. mansoni* ranging from 52.5 to 63.2% and 57.7 to 75.6%, respectively [26, 27].

Faecal samples will be collected to detect intestinal helminths. Duplicate Kato-Katz thick smears will be produced from the stool samples and examined by experienced technicians using light microscopy to determine the egg counts for *S. mansoni* and STH. The numbers of eggs per slide will be used to obtain a measure of the number of helminth eggs per gramme of faeces (EPG). The intensity of the helminth infection will be categorised according to WHO guidelines [28]. A multiplex PCR assay will also be used for simultaneous detection of mixed infections of helminths [29]. Quality control will be performed by re-examining at least 10% randomly selected blood slides, urine filters and Kato-Katz smears by an experienced independent lab scientist. Haemoglobin concentrations will be measured using the Haemocue® haemoglobin analyzer. Duplicate dried blood spots will be shipped to LSHTM for Luminex multiplex immunoassays [30].

### Statistical methods

#### Statistical methods for primary and secondary outcomes {20a}

Infection intensities of positive samples will be calculated as the geometric mean of parasites per microlitre of blood for and geometric mean of eggs per gram of faeces for *S. mansoni*. For *S. haematobium*, infection intensity will be calculated as geometric mean of eggs per 10ml of urine. For pre- and post-intervention time points, the chi-square test will be used to compare proportions (prevalence) of malaria infection and prevalence of anaemia between the intervention and control groups. Similarly, for each time point, the Student *t*-test will be used to compare geometric mean parasite counts and mean haemoglobin concentrations between the intervention and control groups. The repeated measures analysis of variance will be used to compare geometric mean parasite counts and mean haemoglobin concentrations where repeated measurements for pre- and post-intervention time points are compared. All statistical tests will be two-sided at a significance of alpha < 0.05.

Qualitative data will be managed by NVivo software version 12.0 and analysis will be done by initially coding the main themes that emerge from the transcribed texts. The themes will subsequently be sorted and collated into categories and sub-categories. Hypotheses and concepts will be developed inductively from the themes. The findings of the qualitative and quantitative data addressing similar concepts will be triangulated in the final report.

#### Interim analyses {21b}

There are no anticipated problems that are detrimental to the study participants; hence, no interim analyses will be conducted.

#### Methods for additional analyses (e.g. subgroup analyses) {20b}

There are no anticipated needs for additional analyses outside what have been described under the statistical analysis plan.

#### Methods in analysis to handle protocol non-adherence and any statistical methods to handle missing data {20c}

The intention-to-treat (ITT) method will be used to analyse data obtained from all study participants assigned to a group by randomisation. Data from study participants who complete all four cycles of SMC will be analysed using per protocol method. Participants who have only a pre- or post-intervention data will be excluded from the full analysis set.

#### Plans to give access to the full protocol, participant-level data and statistical code {31c}

Public access will be granted to the full protocol, participant-level dataset and statistical code.

### Oversight and monitoring

#### Composition of the coordinating centre and trial steering committee {5d}

**Table:** 

**Collaborating centre:**	University of Thies,
	Cite Malick Sy, PO Box 967, Thies
	Senegal
**Study site:**	Saraya district, Kedougou region, Senegal
**Sponsor:**	London School of Hygiene & Tropical Medicine
	Keppel Street, London WC1E 7HT, UK
**Principal investigator:**	Dr Muhammed Afolabi
	Department of Disease Control
	London School of Hygiene & Tropical Medicine,
	Keppel Street, London WC1E 7HT
	E-mail: Muhammed.Afolabi@lshtm.ac.uk
**Co-investigators:**	Professor Brian Greenwood
	Department of Disease Control
	London School of Hygiene & Tropical Medicine,
	Keppel Street, London WC1E 7HT
	E-mail: Brian.Greenwood@lshtm.ac.uk
	Professor Jean Louis Ndiaye
	Service de Parasitologie Mycologie
	Departement Biologie Medicale
	UFR Santé, Université de Thies, Thies, Senegal
	E-mail: jlndiaye@univ-thies.sn
	Professor Doudou Sow
	Ancien interne des hôpitaux
	Service de Parasitologie-Mycologie,
	UFR des Sciences de la Santé
	Université Gaston Berger de Saint-Louis
	E-mail: doudsow@yahoo.fr; doudou.sow@ugb.edu.sn
**Collaborators**:	Dr Babacar Gueye
	Head of Diseases Control Unit
	Ministère de la Santé et de l'Action Sociale, Senegal
	E-mail: bbcar137@gmail.com
	Dr Doudou Sene
	Coordinator, National Malaria Control Programme
	Ministère de la Santé et de l'Action Sociale, Senegal
	E-mail: drdocsene@yahoo.fr
	Dr Ndeye M’backé Kane
	National Coordinator, NTD Control Programme
	Ministère de la Santé et de l'Action Sociale, Senegal
	E-mail: mbackekane2007@yahoo.fr
	Dr Boubacar Diop
	National Coordinator, Schistosomiasis & STH Control Programme
	Ministère de la Santé et de l'Action Sociale, Senegal
	E-mail: docbocardiop@yahoo.fr
	Dr Baba Camara
	District Medical Officer
	Saraya District Health Centre
	Saraya, Senegal
	E-mail: marababs28@gmail.com

#### Composition of the data monitoring committee, its role and reporting structure {21a}

Given that the trial drugs are licensed products with acceptable safety profiles in more than 30 million children in Central and West Africa [31], a formal Data Monitoring Committee (DMC) will not be constituted. Nevertheless, if a suspected drug-related adverse event is reported to the study staff, the event will be investigated by a local safety monitor who will be an experienced paediatrician based in Senegal. S/he will visit the child’s home to examine the child and interview the parents/caregivers, meet with health staff and check health centre records and complete an adverse event form and report. The local safety monitor will report the findings to the sponsor of the study. If the safety monitor, investigators or sponsors have any safety concerns (e.g. death or severe generalised skin reactions), an independent panel of three malaria and NTD researchers will review severe and serious suspected adverse drug reactions and data from all inpatient records for children admitted to the hospital for at least 24 h within one month of SMC administration to evaluate severity and possible relationship with the study drugs. The description of how SAEs will be recorded is provided below.

#### Adverse event reporting and harms {22}

For the primary endpoint on safety and tolerability of the combined drugs, active surveillance will be used for the collection and collation of adverse drug reactions and adverse events such as vomiting, skin rash, body weakness, etc. Trained field workers, under the supervision of the investigators, will visit all enrolled children irrespective of the study arms, daily at home starting from the evening of days 0 till 3 days after completion of the first cycle of SMC course, to collect adverse events and adverse drug reactions using a purpose-designed electronic diary card. In the context of a pandemic, if travel or home visit is not possible, these follow-up visits may be done by video or telephone call. In the event that the field worker finds any grade 3 (severe) solicited symptoms, the participant will be brought to the nearest health centre or health post for examination by a study clinician.

During the field worker visits, the children’s parents/caregivers will be asked retrospectively if any medical event that might be a serious adverse event (SAE) occurred since the last visit and this information will be recorded in the diary card. Unreported SAEs detected in this way will be investigated and reported by the PI or delegate on the corresponding SAE reporting form. If a study participant is reported to be unwell at the time of a visit, the field worker will advise the parent/caregiver to report to Saraya health centre or the nearest health post, where a member of the study team will be notified for follow-up. In the event that a study participant is seriously ill, appropriate referral and transfer to the nearest referral hospital will be arranged for serious illness following review by telephone or clinical review in person.

A passive surveillance system will also be implemented during the subsequent SMC cycles to complement the system described above. All study participants presenting for admission through the outpatient or emergency departments of Saraya health centre and health posts in the study villages will be evaluated. During the hospitalisation, the participant’s course of illness will be monitored to capture the symptoms and signs indicative of a severe adverse event. A blood sample for the evaluation of malaria parasites will be taken for all children attending a clinic who are reported to have had a fever within 24 h of presentation or have a measured axillary temperature of ≥37.5°C. Other possible sources of fever will also be investigated. A malaria rapid diagnosis test (RDT) will be performed to guide immediate patient management.

The investigators will evaluate any safety information that is spontaneously reported by a study participant beyond the time frame specified in the protocol. The investigators will monitor and analyse the study data including all adverse events and clinical laboratory data as they become available and will make determinations regarding the severity of the adverse events and their relation to a study drug. The investigator or clinical designee will review both adverse drug reactions and other adverse events to ensure prompt and complete identification of all events that require expedited reporting as serious adverse events.

#### Setting up of surveillance teams

A surveillance system will be established with the health centres and health posts in the study areas, before the start of the malaria transmission period, to detect and record adverse reactions and adverse events that may be related to SMC and anthelminthic drugs as implemented successfully in previous SMC campaigns in several districts in Senegal [18].

Training workshops will be conducted for staff at the health facilities on how to recognise and manage adverse drug reactions and how to document and report any adverse events suspected to be drug related. Severe skin reactions and signs of liver disease, and severe vomiting, will be highlighted as adverse events of special interest.

A purpose-designed leaflet with pictorial illustrations of the most common features of adverse drug reactions to SP, AQ, ALB and PZQ will be prepared and used for the training sessions. Copies will be distributed to all health centres and health posts in the study areas and all staff will be encouraged to use the leaflets. A reminder system will be implemented using short text messages (SMS) delivered via mobile telephones to all health centre staff reminding them to regularly look out for adverse events and to report by SMS any serious events. A phone credit will be provided to the staff as an incentive, and they will be contacted after each SMC round to confirm whether they have observed any adverse events. The health post staff will be linked to a member of the study team who will contact them by phone or in person throughout the study to maintain contact, ask about any problems, give support and advice and remind them about study procedures using a standardised list of reminders. During the period of delivery of SMC and anthelminthic drugs, unannounced supervisory visits will be made to each health centre by either the PI or a designated person to monitor compliance with adverse event reporting. A digital camera will be provided to document the occurrence of any skin rash.

During household visits to deliver the first monthly round of SMC and anthelminthic drugs, trained research assistants will explain to the parent/caregiver the purpose of the combined treatments again in simple language stating that all drugs can cause side effects in some children; that AQ may cause vomiting, and SP may sometimes cause skin reactions; ALB may sometimes cause fever and rarely sore throat; and PZQ may cause dizziness and headache but that severe problems are rare with these drugs. The parents/carers will be informed that if side effects of SMC and helminth treatments are suspected, the child should be taken to the nearest health centre or health post without delay. Trained research assistants will visit each child 1 month after each round of SMC cycle to check that there are no severe reactions to the previous treatment and to give the next round of treatment.

After each transmission period, all inpatient records from the health centres and posts will be collated for retrospective analysis for any adverse events that may be drug-related but not reported. Deaths will be investigated by verbal autopsy and reviewed for possible association with SMC and anthelminthic drugs. Serious adverse events will be reported to the national regulatory authority in Senegal within 24 h of becoming aware of it.

#### Frequency and plans for auditing trial conduct {23}

The Trial Steering Group and Ethics Committee will meet to review conduct throughout the trial period.

#### Plans for communicating important protocol amendments to relevant parties (e.g. trial participants, ethical committees) {25}

Amendments of the protocol during the course of the trial will be submitted to the Research Ethics Committees of the London School of Hygiene & Tropical Medicine and the Comité National d’Ethique pour la Recherche en Santé (CNERS) in Senegal, for review and approval. The study participants and their parents/caregivers will also be informed about the changes in the study.

*Dissemination plans {31a}*

Findings of this study will be disseminated through meetings with community and religious leaders in the study site, managers at the national control programmes for malaria and neglected tropical diseases in Senegal and other stakeholders. The findings will also be presented at scientific meetings and published in a peer-reviewed journal.

## Discussion

Malaria and helminths are two parasitic diseases commonly co-existing among pre-school and school-age children in SSA [1, 3]. Following the London Declaration on NTDs in 2012, anthelminthic drugs were donated generously by pharmaceutical companies to meet the WHO target of 75% treatment coverage in pre-school and school-age children in all affected countries by 2020. Recent reports have shown that very few countries have met this target [32], and many have not demonstrated the expected impact on the prevalence of parasite infections. This shortfall is due to a combination of poor programmatic coverage, sub-optimal adherence and the exposure of parasites to sub-therapeutic drug concentrations [33].

On the other hand, seasonal malaria chemoprevention (SMC), which was recommended in the same year as the London declaration on NTDs (2012) by the WHO, has achieved more than 75% treatment coverage and prevented 75–85% cases of uncomplicated and severe malaria in children aged below 5 years in some situations [34]. The extension of SMC to children aged 5–10 years has been implemented successfully in Senegal [35] and is being considered in other West African countries. This encouraging development supports the need to explore new strategies involving integration of NTD control efforts with other successful platforms such as SMC. This integrated approach has the potential to align NTD and malaria control and elimination efforts with the SDG of ending the diseases of poverty and promote health and well-being of those at risk.

The global trend towards integrated, non-disease-specific approaches to controlling co-endemic diseases such as malaria and helminths has become an increasingly recognised strategy, but limited information exists on the safety, feasibility and effectiveness of combining SMC with helminth control at the community level [36, 37]. SMC involves administration of full treatment courses of antimalarial medications at monthly intervals during the malaria transmission to prevent malaria illness, with the objective of maintaining the therapeutic anti-malaria drug concentrations in the blood throughout the period of greatest malaria risk [31]. The approved preventive chemotherapy for mass drug administration (MDA) for STH and schistosomes (albendazole [ALB] and praziquantel [PZQ]) respectively, and the SMC drugs (AQ plus SP) work well with minimal side effects when used separately [35, 38]. Given that 4-aminoquinoline drugs are reported to have antagonistic pharmacokinetic interactions with praziquantel [39], the SMC partner drugs, AQ (a 4-aminoquinoline derivative) and SP will not be administered at the same time with anthelminthic drugs (albendazole and praziquantel) in this study. Because albendazole and praziquantel have a short half-life of 8.5 h and <2h respectively [22], anthelminthic drugs (praziquantel and albendazole) will be administered a day before the SMC drugs (AQ and SP) providing ample opportunity to objectively assess the tolerability of the different drug regimen.

The limitations of this study include the inability to mask the study drugs from parents and their children. Nevertheless, the use of control medications will ensure all study children receive equal number of medications and this could minimise bias due to unmasking. Also, the prevalence of malaria and STH is low in the study area and this may make it challenging to determine the effectiveness of the combined administration of SMC and anthelminthic drugs among the study participants but the study will provide important information on the safety and tolerability of administering SMC and antihelminthincs through a common delivery system. Measurement of efficacy is likely to require a larger study.

Overall, this study may provide evidence to boost the public health recommendations for combined malaria and helminth control. If successful, this project will reinforce the empirical evidence that the future direction of health care systems in developing countries should be comprehensive health management rather than vertical management of a single disease.

### Trial status

This paper is based on protocol version 2.0, dated 8 April 2022. Recruitment of the study participants will coincide with the commencement of the first cycle of SMC scheduled by the Senegal Ministry of Health for 17 June 2022. The recruitment was completed on 21 June 2022, and the follow-up of the enrolled participants will continue until 30 November 2022.

## Data Availability

The principal investigator and designated study personnel will have access to the final trial dataset. We disclose no contractual agreements that limit such access for the investigators.

## Abbreviations

AE: Adverse events
ALB: Albendazole
AQ: Amodiaquine
Hb: Haemoglobin
PCR: Polymerase chain reaction
PI: Principal investigator
PZQ: Praziquantel
SAE: Serious adverse event
SD: Standard deviation
SMC: Seasonal malaria chemoprevention
SP: Sulphadoxine-pyrimethamine
STH: Soil-transmitted helminths
